# Dietary Habits and Nutritional Status of Different Population Groups in Relation to Lifestyle Factors and Nutritional Knowledge

**DOI:** 10.3390/nu15214609

**Published:** 2023-10-30

**Authors:** Agata Wawrzyniak, Barbara Pietruszka

**Affiliations:** Department of Human Nutrition, Institute of Human Nutrition Sciences, Warsaw University of Life Sciences (SGGW-WULS), 02-787 Warsaw, Poland; barbara_pietruszka@sggw.edu.pl

Proper nutrition and a balanced diet are factors that influence the growth and development of children and adolescents, as well as body weight and health throughout life. Poor nutritional status resulting from unhealthy eating can cause many diseases, including obesity and other chronic non-communicable diseases such as cardiovascular diseases, cancers and diabetes [1,2]. According to statistics, the above-mentioned diseases are responsible for approximately 80% of premature deaths. Knowledge about the degree of implementation of current dietary and lifestyle recommendations in various groups allows us to detect possible errors in this area and prevent irregularities in the nutritional status [3], hence the subject of this Special Issue of *Nutrients* entitled “Dietary Habits and Nutritional Status of Different Population Groups in Relation to Lifestyle Factors and Nutritional Knowledge”. This Special Issue comprises five articles covering various very important thematic areas, and each of them adopts a different research approach. The articles discuss the issues of collecting data on nutritional competences, the impact of various factors, including health status, on the knowledge and implementation of dietary recommendations, as well as the relationship between knowledge of nutritional principles and their implementation. There is no doubt that all the research presented in this Special Issue, as well as the scientific literature discussed in the articles, is crucial to understanding the impact of dietary habits and nutritional status in connection with lifestyle factors and nutritional knowledge.

The article “Food Literacy Scale: Validation through Exploratory and Confirmatory Factor Analysis in a Sample of Portuguese University Students [2]” discusses the methodological aspects of measuring eating skills. The appropriate methodology for examining dietary behaviors and choices, including choices of food with health-promoting properties, is extremely important, especially in the analysis of their connections with nutritional knowledge. Therefore, researchers undertook the development and validation of a scale for assessing food literacy. The validated scale consisted of items divided into three areas: literacy about the nutritional composition of food, literacy about food labeling and choices, and ability to use healthy eating practices. This scale may be used as a valuable research tool in the future.

Research results indicate that nutritional knowledge is not always applicable in practice [4,5,6,7]. This observation is reflected in two studies within this Special Issue, one examining the consumption of sweetened drinks in place of water, and the other focusing on ultra-processed foods. At the same time, the authors analyzed the factors related to the consumption of these types of products.

In the article “Knowing is not Doing: A Qualitative Study of Parental Views on Family Beverage Choice [8]” the authors presented the problem of excessive consumption of sweet drinks in the family, which may be harmful to children’s health. This article draws attention to the need for scalable family interventions promoting water consumption instead of high-energy drinks. By identifying families in which children consumed excessive amounts of sugar-sweetened beverages and/or fruit juice, the authors conducted a study using parent interviews to develop a scaled health system-based intervention targeting health-promoting beverage choices. The authors proved that knowledge is not enough to change health-promoting behavior. Moreover, beverage interventions must first make water consumption particularly attractive. Implementing this type of intervention could provide a higher level of care, and technology would reduce the burden on doctors and parents. In turn, the article “Association between the Consumption of Ultra-Processed foods and Sociodemographic Characteristics in Brazilian Adolescents [9]” concerned the consumption of ultra-processed food, which may be associated with a number of negative health effects. Young people are currently consumers of ultra-processed products, especially in highly developed countries. The authors of the study confirmed that young people from families with a higher economic level, females, Caucasians, and those living in cities (but outside the capital) were exposed to increased consumption of ultra-processed products. Therefore, unprocessed or minimally processed food should be particularly promoted, including in government programs. Nutrition education should promote the implementation of dietary recommendations and support the right to healthy and nutrient-rich food.

Nutritional knowledge and knowledge about the health effects of improper nutrition may also influence the decision to change one’s diet, including, among other things, a transition to vegetarianism. Plant-based diets, including vegan diets, offer numerous health benefits, including maintaining a healthy body weight [10], reducing the risk of various diseases, such as diabetes [10,11], and lowering the incidence of and mortality from conditions like ischemic heart disease and cancer [12]. People consuming plant-based diets also emphasized the following as being important: positive feelings related to moral attitude, an increased sense of belonging (to the vegetarian community) and a lower negative impact on the environment [13]. The authors of the article “Comparison of the Health Status of Vegetarians and Omnivores Based on Biochemical Blood Tests, Body Composition Analysis and Quality of Nutrition [14]” confirmed in their research that the health status of vegetarians was better compared to omnivores. Additionally, vegetarians who followed dietary recommendations were also more physically active and led healthier lifestyles; however, lower levels of vitamin B_12_ and vitamin D and increased levels of homocysteine were detected in the blood of people on a vegetarian diet.

In a recent article entitled “Examining Nutrition Knowledge, Skills and Eating Behaviors in People with Severe Mental Illness: A Cross-Sectional Comparison among Psychiatric Inpatients, Outpatients and Healthy Adults [15]”, important issues related to the nutrition, nutritional status and nutritional knowledge of chronically ill people were discussed. Patients with severe mental illness (SMI) are often underweight, overweight, or have metabolic syndrome due to their illness and/or unhealthy lifestyle. In the conducted research, patients with SMI were diagnosed with poorer nutritional status and poorer adherence to the principles of proper nutrition; however, the levels of nutritional knowledge, skills in preparing and eating meals, and motivation to eat healthily were not significantly lower than in healthy people. Therefore, to maintain better health, SMI patients should be included in long-term nutritional support programs.

Effective explanation of all these relationships will allow for the preparation of effective intervention strategies in the future using the research methods used in the above studies. These findings will not only help in the development of additional evidence-based dietary guidelines, but may also help health care professionals and dietitians to educate people from various population groups and encourage them to adopt healthy eating behaviors.

## References

[1] Kabir A., Miah S., Islam A. (2018). Factors Influencing Eating Behavior and Dietary Intake among Resident Students in a Public University in Bangladesh: A Qualitative Study. PLoS ONE.

[2] Guiné R.P.F., Florença S.G., Aparício G., Cardoso A.P., Ferreira M. (2023). Food Literacy Scale: Validation through Exploratory and Confirmatory Factor Analysis in a Sample of Portuguese University Students. Nutrients.

[3] World Health Organization (WHO) (2022). Noncommunicable Diseases. https://www.who.int/news-room/fact-sheets/detail/noncommunicable-diseases.

[4] Tufail S., Ahmed A., Kanwal R., Malik A., Noor Z., Mushtaq Q.U.A. (2020). Dietary habits and knowledge of nutritional requirements of students of a private medical college. Pak. Armed Forces Med. J..

[5] Akinmoladun O., Oluyede O., Femi F., Olaitan O., Nesamvuni C. (2021). Association between nutrition knowledge, lifestyle, dietary practices and nutritional status among civil servants in western Nigeria. Afr. J. Food Agric. Nutr. Dev..

[6] Dorado M.A.G.M., Racca A.P. (2019). Relationship of Knowledge on Healthy Lifestyle to Dietary Practices and Physical Activity as Moderated by Age. ISC.

[7] Issahaku I., Alhassan M. (2021). Nutrition knowledge, dietary practices and nutritional status of non-academic staff at the Tamale campus of University for Development Studies. Heliyon.

[8] Newman C.M., Zoellner J., Schwartz M.B., Peña J., Wiseman K.D., Skelton J.A., Shin T.M., Lewis K.H. (2023). Knowing Is Not Doing: A Qualitative Study of Parental Views on Family Beverage Choice. Nutrients.

[9] Gonçalves H.V.B., Batista L.S., de Amorim A.L.B., Bandoni D.H. (2023). Association between Consumption of Ultra-Processed Foods and Sociodemographic Characteristics in Brazilian Adolescents. Nutrients.

[10] Termannsen A.D., Clemmensen K.K.B., Thomsen J.M., Nørgaard O., Díaz L.J., Torekov S.S., Quist J.S., Faerch K. (2022). Effects of vegan diets on cardiometabolic health: A systematic review and meta-analysis of randomized controlled trials. Obes. Rev..

[11] Olfert M.D., Wattick R.A. (2018). Vegetarian Diets and the Risk of Diabetes. Curr. Diabetes Rep..

[12] Dinu M., Abbate R., Gensini G.F., Casini A., Sofi F. (2017). Vegetarian, vegan diets and multiple health outcomes: A systematic review with meta-analysis of observational studies. Crit. Rev. Food Sci. Nutr..

[13] Hargreaves S.M., Raposo A., Saraiva A., Zandonadi R.P. (2021). Vegetarian Diet: An Overview through the Perspective of Quality of Life Domains. Int. J. Environ. Res. Public Health.

[14] Jedut P., Glibowski P., Skrzypek M. (2023). Comparison of the Health Status of Vegetarians and Omnivores Based on Biochemical Blood Tests, Body Composition Analysis and Quality of Nutrition. Nutrients.

[15] Mötteli S., Provaznikova B., Vetter S., Jäger M., Seifritz E., Hotzy F. (2023). Examining Nutrition Knowledge, Skills, and Eating Behaviours in People with Severe Mental Illness: A Cross-Sectional Comparison among Psychiatric Inpatients, Outpatients, and Healthy Adults. Nutrients.

